# lncRNA ZFAS1 promotes lung fibroblast-to-myofibroblast transition and ferroptosis via functioning as a ceRNA through miR-150-5p/SLC38A1 axis

**DOI:** 10.18632/aging.103176

**Published:** 2020-05-26

**Authors:** Yanni Yang, Wenlin Tai, Nihong Lu, Ting Li, Yongjun Liu, Wenjuan Wu, Zhengkun Li, Lin Pu, Xiaoyuan Zhao, Tao Zhang, Zhaoxing Dong

**Affiliations:** 1 Department of Ophthalmology, The Second Affiliated Hospital of Kunming Medical University, Kunming 650101, Yunnan, China; 2 Department of Clinical Laboratory, Yunnan Molecular Diagnostic Center, The Second Affiliated Hospital of Kunming Medical University, Kunming 650101, Yunnan, China; 3 Department of Respiratory, The Third People’s Hospital of Kunming, Kunming 650041, Yunnan, China; 4 Department of Respiratory, The Second Affiliated Hospital of Kunming Medical University, Kunming 650101, Yunnan, China

**Keywords:** pulmonary fibrosis, lipid peroxidation, lncRNA ZFAS1, fibroblast activation, miR-150-5p/SLC38A1

## Abstract

Pulmonary fibrosis (PF) is a lethal fibrotic lung disease. The role of lncRNAs in multiple diseases has been confirmed, but the role and mechanism of lncRNA zinc finger antisense 1 (ZFAS1) in the progression of PF need to be elucidated further. Here, we found that lncRNA ZFAS1 was upregulated in bleomycin (BLM)-induced PF rats lung tissues and transforming growth factor-β1 (TGF-β1)-treated HFL1 cells, and positively correlated with the expression of solute carrier family 38 member 1 (SLC38A1), which is an important regulator of lipid peroxidation. Moreover, knockdown of lncRNA ZFAS1 significantly alleviated TGF-β1-induced fibroblast activation, inflammation and lipid peroxidation. *In vivo* experiments showed that inhibition of lncRNA ZFAS1 abolished BLM-induced lipid peroxidation and PF development. Mechanistically, silencing of lncRNA ZFAS1 attenuated ferroptosis and PF progression by lncRNA ZFAS1 acting as a competing endogenous RNA (ceRNA) and sponging miR-150-5p to downregulate SLC38A1 expression. Collectively, our studies demonstrated the role of the lncRNA ZFAS1/miR-150-5p/SLC38A1 axis in the progression of PF, and may provide a new biomarker for the treatment of PF patients.

## INTRODUCTION

Pulmonary fibrosis (PF) is a chronic, progressive and irreversible pulmonary interstitial tissue heterogeneous disease that is characterized by persistent alveolar epithelial injury [1]. In recent years, several studies found that the development of PF was related to the activation of fibroblast-to-myofibroblast transition (FMT), abnormal tissue remodeling, immune response and excessive extracellular matrix (ECM) deposition [2]. Moreover, the process of FMT is a prominent pathway leading to ECM deposition, and inhibiting the differentiation of myofibroblasts was demonstrated as an effective way to prevent PF [3, 4]. Clinical studies have confirmed that drugs including hormones, non-hormone immunosuppressants, and antifibrotic, anticytokine and immunomodulatory agents, had no significant effect on the treatment of PF, and there was no effective treatment found for PF patients [5, 6]. Although many studies have confirmed that FMT activation, ECM deposition and inflammation were involved in the progression of PF, the underlying mechanism of FMT activation in promoting the progression of PF remains largely undetermined.

Accumulating evidence has confirmed that long non-coding RNAs (lncRNAs) are involved in many pathophysiological processes of PF, including cell proliferation, migration, epithelial-mesenchymal transition (EMT), and immunoregulation [7, 8]. For example, Song et al. found that the abnormal expression of lncRNA and protein-coding gene was associated with the progression of PF through as competing endogenous RNA (ceRNA) [9]. Wu et al. showed that lncRNA CHRF promoted the progression of PF by downregulating the inhibitory effect of miR-489 on the expression of MyD88 and Smad [10]. Zhao et al. reported that lncRNA PFAR acted as ceRNA to promote the FMT process and myofibroblast differentiation by regulating the miR-138/YAP1 axis [11]. In addition, lncRNA zinc finger antisense 1 (lncRNA ZFAS1) was originally identified as a novel tumor-related lncRNA by upregulating cell proliferation, migration, and EMT [12, 13]. Recently, upregulation of lncRNA ZFAS1 was demonstrated to induce EMT and ECM deposition by promoting the expression of ZEB2 [14], suggesting the potential involvement of lncRNA ZFAS1 in PF progression. However, the role of lncRNA ZFAS1 in the progression of PF requires further study and confirmation.

Ferroptosis is a newly characterized iron-dependent form of non-apoptotic regulated cell death triggered by lipid reactive oxygen species (ROS). Interestingly, several studies found that ROS levels were upregulated in the process of FMT induced by TGF-β1 [15, 16], which was triggered by inflammatory cytokine secretion in PF [17–19]. Moreover, previous studies confirmed that iron overload could lead to PF, which is related to the increase in lipid peroxidation and the decrease in glutathione peroxidase 4 (GPX4) activity in lung tissues [20]. For example, upregulation of GPX4 decreased myofibroblast differentiation, ROS levels and ferroptosis in the TGF-β1-induced PF cell model [21]. Furthermore, increasing evidence has confirmed that glutamine metabolism contributes to the formation of oxidizable lipids, which could lead to ferroptosis [22, 23], and the level of glutathione in PF lung tissues was downregulated [24, 25]. In addition, SLC38A1 is an important regulator of glutamine uptake and metabolism in lipid peroxidation [26]. Therefore, we speculated that ferroptosis plays an important in the progression of PF, and the role of SLC38A1 in PF through ferroptosis regulation remains elusive.

In this study, the effect of lncRNA ZFAS1 on the process of FMT and ferroptosis was determined. Mechanistically, we further to explore whether lncRNA ZFAS1 regulates fibroblast activation and lipid peroxidation via sponging miR-150-5p and regulating SLC38A1 in the progression of PF. Overall, our study will provide vital theoretical evidence for explaining the mechanisms of the lncRNA ZFAS1/miR-150-5p/SLC38A1 axis in PF progression, and will simultaneously provide a new biomarker and target for the diagnosis and treatment of PF.

## RESULTS

### Upregulation of lncRNA ZFAS1 in PF is positively correlated with SLC38A1 expression

**Figure 1:**
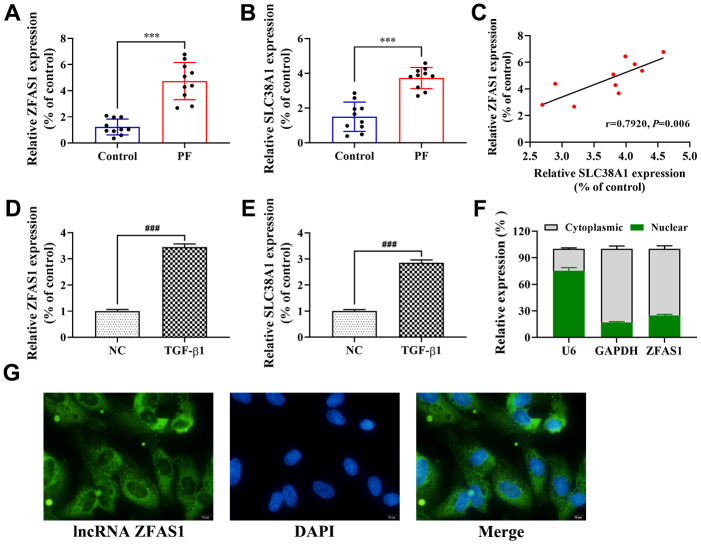
**Upregulation of lncRNA ZFAS1 in PF is positively correlated with SLC38A1 expression.** (**A**, **B**) RT-qPCR was performed to detect the expression of lncRNA ZFAS1 and SLC38A1 in lung tissues; (**C**) Spearman analysis was used to analyze the association between lncRNA ZFAS1 and SLC38A1 expression in the lung tissues of BLM-induced pulmonary fibrosis cases; (**D**, **E**) The expression of lncRNA ZFAS1 and SLC38A1 in HFL1 cells treated with TGF-β1 or control were determined by RT-qPCR; (**F**) RT-qPCR was used to measure the expression of lncRNA ZFAS1 in either the nucleus or cytoplasm of HFL1 cells; (**G**) FISH was performed to evaluate the location of endogenous lncRNA ZFAS1 (green) in HFL1 cells, U6 and GAPDH were used as nuclear and cytoplasmic localization markers, respectively. DNA (blue) was stained with DAPI. ^***^P<0.001, compared with the control group; ^###^P<0.001, compared with the NC group.

A previous studies showed that an abnormally expressed lncRNA was involved in the development and progression of PF by regulating a downstream gene [11]. In this study, the expression of lncRNA ZFAS1 and SLC38A1 in lung tissues was determined by RT-qPCR. As shown in Figure 1A, 1B, lncRNA ZFAS1 and SLC38A1 mRNA were highly expressed in lung tissues of the PF rat model induced by BLM compared with the control group (both P<0.001). Moreover, Spearman’s correlation analysis revealed a remarkably positive correlation between lncRNA ZFAS1 expression and SLC38A1 expression in lung tissues of BLM-induced PF rat model (r=0.792, P<0.01, Figure 1C). Consistent with the *in vivo* studies, the expression levels of lncRNA ZFAS1 and SLC38A1 were higher in TGF-β1-treated HFL1 cells than in the NC group (both P<0.001, Figure 1D, 1E). Furthermore, we attempted to evaluate the subcellular location of lncRNA ZFAS1 in HFL1 cells. The FISH analysis results showed that lncRNA ZFAS1 was mainly distributed in the cytoplasm (Figure 1G). Similarly, RT-qPCR showed that the lncRNA ZFAS1 transcript was preferentially localized in the cytoplasm than in the nucleus (Figure 1F). Taken together, the results showed that lncRNA ZFAS1 was upregulated in PF and positively correlated with SLC38A1, which indicated that overexpression of lncRNA ZFAS1 and SLC38A1 may play an important role in regulating the progression of PF.

### Knockdown of lncRNA ZFAS1 inhibits the FMT process in TGF-β1-induced HFL1 cells

**Figure 2:**
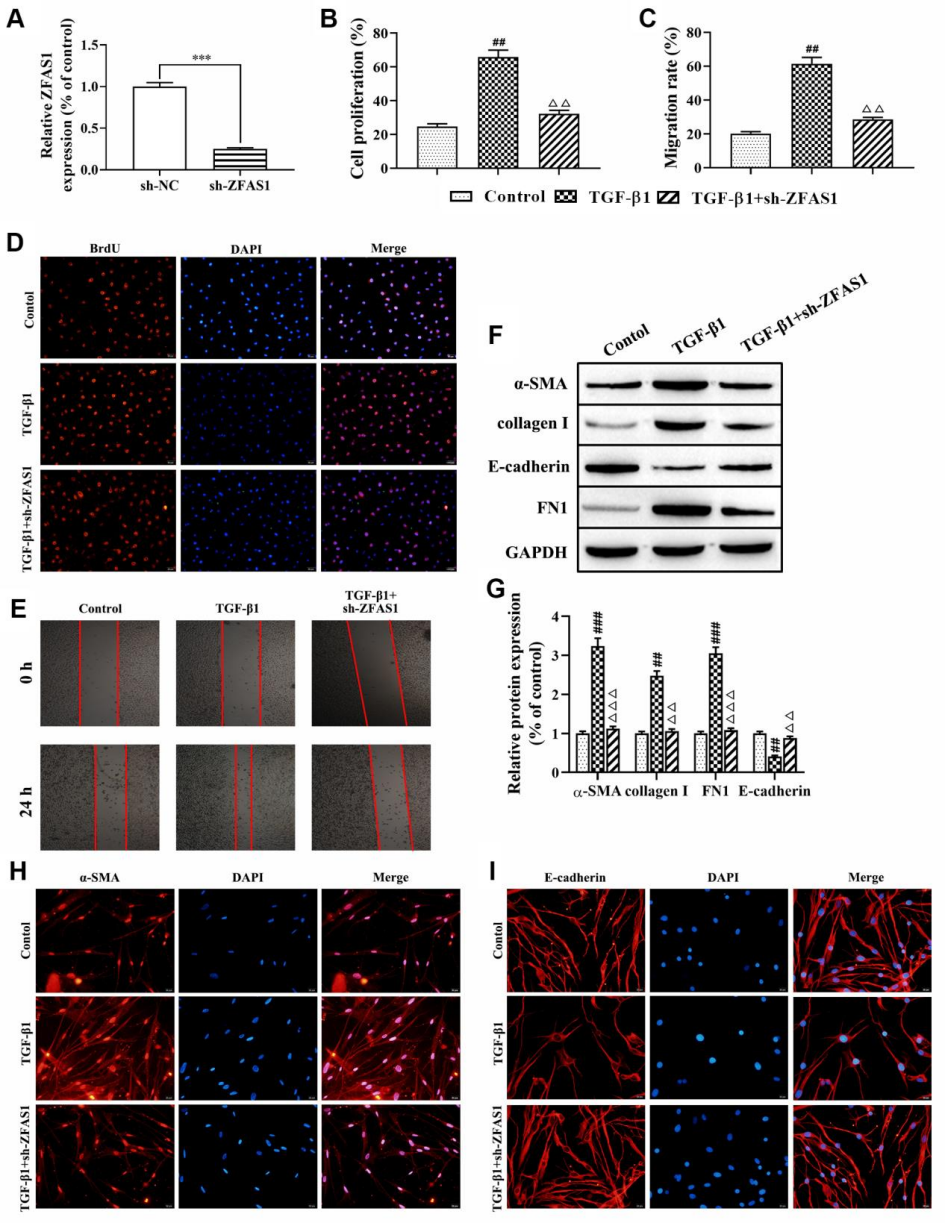
**Knockdown of lncRNA ZFAS1 inhibits the FMT process in TGF-β1-induced HFL1 cells.** (**A**) The expression of lncRNA ZFAS1 in HFL1 cells transfected with lncRNA ZFAS1 shRNA was determined by RT-qPCR; (**B**–**D**) BrdU staining was applied to test cell viability; (**C**–**E**) The migration ability of HFL1 cells was measured by wound healing assay; (**F**, **G**) Western blot was performed to detect the expression levels of E-cadherin, collagen I, FN1 and the FMT marker α-SMA; (**H**, **I**) Immunofluorescence staining was applied to evaluate the expression of α-SMA and E-cadherin in HFL1 cells. ^***^P<0.001, compared with the sh-NC group; ^##^P<0.01, ^###^P<0.001, compared with the control group; ^ΔΔ^P<0.01, ^ΔΔΔ^P<0.001, compared with the TGF-β1-treated group.

Accumulating evidence has confirmed that FMT is closely related to the development of PF [2, 27]. First, we transfected HFL1 cells with lncRNA ZFAS1 shRNA and found that lncRNA ZFAS1 expression was significantly decreased compared with that in the negative control (sh-NC) group (P<0.001, Figure 2A). Moreover, BrdU staining and wound healing assay showed that knockdown of lncRNA ZFAS1 significantly restored the TGF-β1-induced proliferation and migration of HFL1 cells (all P<0.01, Figure 2B–2E), while no significant difference was observed between the TGF-β1+sh-ZFAS1 group and the control group. Furthermore, the role of lncRNA ZFAS1 in regulating the TGF-β1-induced FMT process was evaluated. Western blot analysis results showed that knockdown of lncRNA ZFAS1 significantly decreased the protein levels of α-SMA, collagen I, and FN1 (P<0.01, P<0.001, Figure 2F, 2G), but upregulated E-cadherin expression (P<0.01). Similarly, immunofluorescence staining showed that silencing of lncRNA ZFAS1 abolished the inducing effect of TGF-β1 treatment on the expression of α-SMA (Figure 2H), but promoted the expression of E-cadherin (Figure 2I). Overall, knockdown of lncRNA ZFAS1 significantly attenuated TGF-β1-induced FMT process *in vitro*.

### Knockdown of lncRNA ZFAS1 reduces inflammatory cytokine secretion, ROS levels and ferroptosis in HFL1 cells

**Figure 3:**
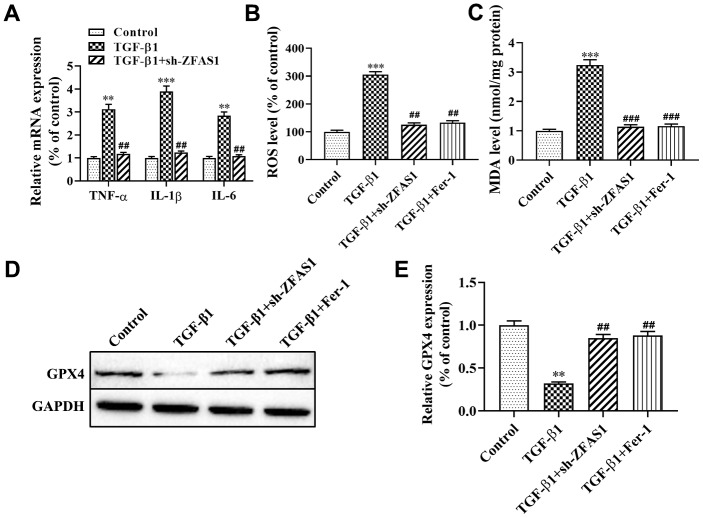
**Knockdown of lncRNA ZFAS1 alleviates TGF-β1-induced ferroptosis in HFL1 cells.** (**A**) RT-qPCR was used to detect the mRNA expression of inflammatory cytokines (TNF-α, IL-1β, and IL-6) in HFL1 cells; (**B**) The level of ROS was measured by ROS kit; (**C**) The MDA content was measured by a lipid peroxidation (MDA) assay kit; (**D**, **E**) Western blot was performed to detect the level of GPX4 protein. ^**^P<0.01, ^***^P<0.001, compared with the control group; ^##^P<0.01, ^###^P<0.001, compared with the TGF-β1 group.

Recent studies have found that lipid peroxidation promotes myofibroblast differentiation and ferroptosis, leading to the progression of PF [16, 21]. In this study, we attempted to evaluate the effect of lncRNA ZFAS1 on inflammation, ROS levels and ferroptosis in HFL1 cells. As shown in Figure 3A, the expression of inflammatory cytokines (TNF-α, IL-6, IL-1β) were significantly upregulated in HFL1 cells when treated with TGF-β1 compared with the control group (P<0.01, P<0.001), while knockdown of lncRNA ZFAS1 decreased its expression (all P<0.01, Figure 3A). Moreover, knockdown of lncRNA ZFAS1 or ferroptosis inhibitor (Fer-1) treatment significantly decreased the TGF-β1-induced high levels of ROS (all P<0.01, Figure 3B). Furthermore, we detected the expression of GPX4 and MDA to evaluate the effect of lncRNA ZFAS1 on lipid peroxidation in ferroptosis. Western blot analysis showed that the protein level of GPX4 was significantly enhanced by Fer-1 administration and lncRNA ZFAS1-silenced in TGF-β1-treated HFL1 cells (P<0.01, Figure 3D, 3E). Moreover, the level of MDA in the TGF-β1-treated group was significantly enhanced compared with that in the control group (P<0.001, Figure 3C), but Fer-1 treatment or lncRNA ZFAS1-slienced abrogated this effect (both P<0.001). In addition, there was no significant difference between the control group and the TGF-β1+sh-ZFAS1 group or TGF-β1+Fer-1 group. Taken together, these results demonstrated that knockdown of lncRNA ZFAS1 decreased the promotion effect of TGF-β1 on inflammation and lipid peroxidation in HFL1 cells.

### miR-150-5p is a target of lncRNA ZFAS1, and miR-150-5p directly targets SLC38A1 in TGF-β1-induced HFL1 cells

**Figure 4:**
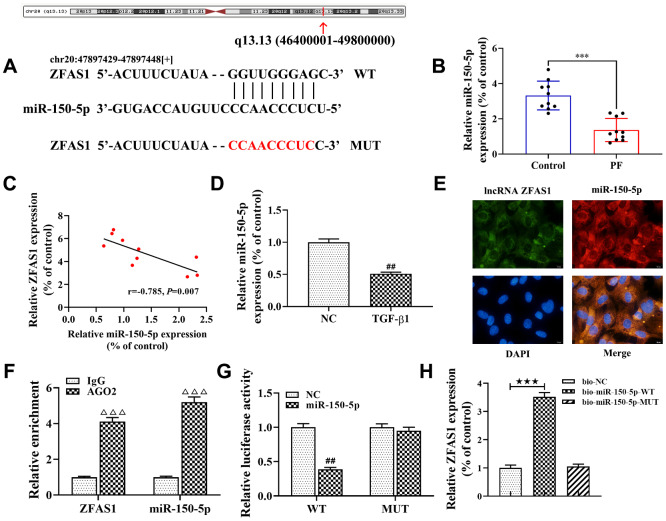
**lncRNA ZFAS1 interacts with and negatively regulates miR-150-5p.** (**A**) Sequence alignment of lncRNA ZFAS1 with the binding sites in miR-150-5p; (**B**) The expression of miR-150-5p in lung tissues was analyzed by RT-qPCR; (**C**) Spearman correlation was used to analyze the association between miR-150-5p and lncRNA ZFAS1 expression in the lung tissues of BLM-induced pulmonary fibrosis; (**D**) RT-qPCR was used to detect the expression of miR-150-5p in HFL1 cells; (**E**) FISH was performed to evaluate the location of endogenous lncRNA ZFAS1 and miR-150-5p in HFL1 cells; (**F**) RT-qPCR was applied to detect the expression of lncRNA ZFAS1 and miR-150-5p in AGO2 pellet; (**G**) dual-luciferase reporter gene was used to verify the targeted relationship between lncRNA ZFAS1 and miR-150-5p; (**H**) RNA pulldown assay showed that biotin labeled miR-150-5p-WT interacted with lncRNA ZFAS1. ^***^P<0.001, compared with the control group; ^##^P<0.01, compared with NC group; ^ΔΔΔ^P<0.001, compared with IgG group; ^★★★^P<0.001, compared with bio-NC group.

Accumulating evidence has confirmed that the lncRNA-miRNA-mRNA network plays an important role in the progression of PF [8, 28]. In this study, bioinformatics tools of StarBase were applied to analyze the potential interaction between lncRNA ZFAS1 and miRNAs. The results showed that miR-150-5p was predicted to be a potential target of lncRNA ZFAS1, and the potential binding site between miR-150-5p and lncRNA ZFAS1 was shown in Figure 4A. Moreover, previous studies have shown that miR-150-5p was associated with fibrosis [29]. As expected, RT-qPCR showed that miR-150-5p was expressed at low levels in the lung tissues of rats with PF compared with the control group (P<0.001, Figure 4B). Similarly, Spearman’s correlation analysis revealed a remarkably negative correlation between the expression of lncRNA ZFAS1 and miR-150-5p in lung tissues of the PF rat model (r=-0.785, P<0.01, Figure 4C). Consistently, the expression of miR-150-5p was down-expressed in TGF-β1-treated HFL1 cells (P<0.01, Figure 4D). In addition, FISH analysis showed that lncRNA ZFAS1 and miR-150-5p co-localized in the cytoplasm of HFL1 cells (Figure 4E). Of note, Chen et al. found that lncRNA ZFAS1 binds to miR-150-5p in an AGO2-dependent manner [30]. As expected, the RIP results also showed that compared with those in the control group (IgG), the expression levels of lncRNA ZFAS1 and miR-150-5p were highly increased in the AGO2 pellet (P<0.001, Figure 4F). Furthermore, the Dual-Luciferase Reporter assay results showed that the luciferase activity of the WT reporter was lower in lncRNA ZFAS1-WT+miR-150-5p group than that in the lncRNA ZFAS1-WT+NC group (P<0.01, Figure 4G), but had no effect on the luciferase activity of the MUT reporter. In addition, RNA pulldown showed that miR-150-5p-WT, but not the mutant one, precipitated lncRNA ZFAS1 (Figure 4H), demonstrating their direct interaction. In light of this, we confirmed that the target gene of lncRNA ZFAS1 was miR-150-5p, and lncRNA ZFAS1 negatively regulated the expression of miR-150-5p.

**Figure 5:**
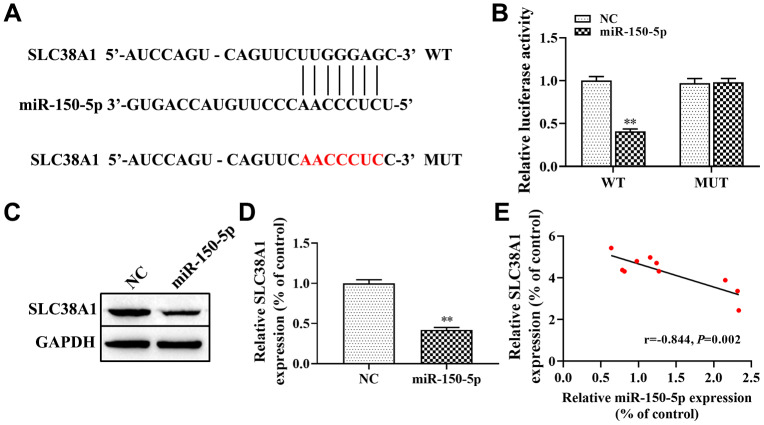
**SLC38A1 is a target gene of miR-150-5p.** (**A**) Bioinformatics analysis indicated the putative binding sites and corresponding mutant region for miR-150-5p within SLC38A1; (**B**) dual-luciferase reporter gene was used to verify the targeted relationship between miR-150-5p and SLC38A1; (**C**, **D**) The effect of miR-150-5p on the expression of SLC38A1 protein was determined by western blot; (**E**) Spearman analysis was used to analyze the association between miR-150-5p and SLC38A1 expression in the lung tissues of BLM-induced pulmonary fibrosis. ^**^P<0.01, compared with the NC group.

In addition, we discovered that miR-150-5p may target SLC38A1 directly as predicted by the Starbase database (Figure 5A). To further confirm that miR-150-5p specifically bind to the 3’UTR of SLC38A1 mRNA to regulate the expression of SLC38A1, we performed dual-luciferase reporter assay. The results showed that the luciferase activity in the SLC38A1-WT+miR-150-5p mimic group was lower than that in the SLC38A1-WT+miR-NC group (P<0.01, Figure 5B), but there was no significant difference when miR-150-5p mimic or NC were co-transfected with SLC38A1-MUT. In addition, we used western blot to test the expression level of SLC38A1 when miR-150-5p mimics were transfected into HFL1 cells treated with TGF-β1. The results indicated that the expression of SLC38A1 was significantly decreased in the miR-150-5p mimic group compared with the NC group (P<0.01, Figure 5C, 5D). Furthermore, Spearman’s correlation analysis revealed a remarkably negative correlation between SLC38A1 expression and miR-150-5p expression in lung tissues of PF rats (r=-0.844, P<0.01, Figure 5E). In conclusion, these results suggest that SLC38A1 was a direct target of miR-150-5p, which negatively regulated SLC38A1 expression.

### lncRNA ZFAS1 regulates FMT and ferroptosis via the miR-150-5p/SLC38A1 axis

**Figure 6:**
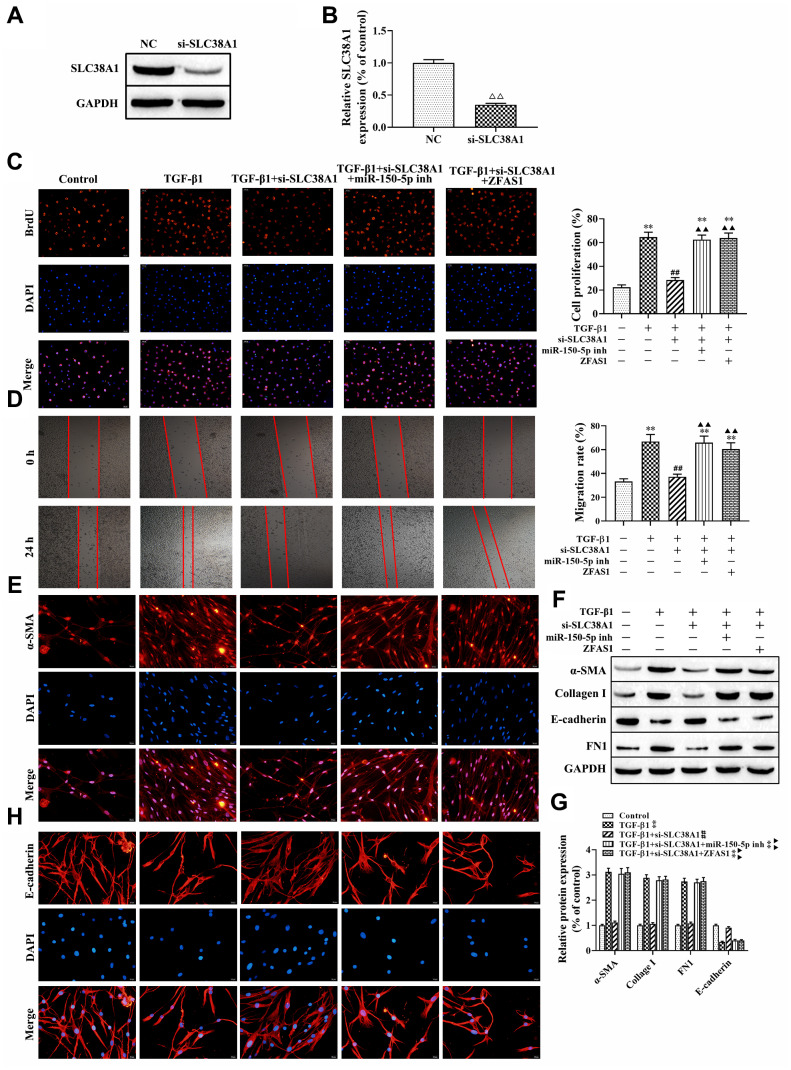
**lncRNA ZFAS1 regulates FMT activation via the miR-150-5p/SLC38A1 axis in HFL1 cells.** (**A**, **B**) Western blot was performed to detect the expression of SLC38A1 in HFL1 cells; (**C**) BrdU staining was applied to analyze cell viability; (**D**) Wound healing assay was used to detect the migration of HFL1 cells; (**E**, **H**) The expression levels of α-SMA and E-cadherin in HFL1 cells were measured by immunofluorescence staining; (**F**, **G**) Western blot was performed to detect the expression levels of E-cadherin, collagen I, FN1 and α-SMA. ^ΔΔ^P<0.01, compared with NC group; ^**^P<0.01, compared with the control group; ^##^P<0.01, compared with the TGF-β1 group; ^▲▲^P<0.01, compared with the TGF-β1+si-SLC38A1 group.

Next, we further to explore the role of the lncRNA ZFAS1/miR-150-5p/SLC38A1 axis in the process of FMT and ferroptosis in the PF cell model induced by TGF-β1. First, we used SLC38A1 shRNA to decrease the expression of SLC38A1 in HFL1 cells, and the transfection efficiency was determined by western blot (P<0.01, Figure 6A, 6B). BrdU staining and wound healing assay showed that SLC38A1 knockdown decreased TGF-β1 induced the cell viability and migration (all P<0.01, Figure 6C, 6D). However, there was no significant difference between the TGF-β1 treatment only group and the rescue group (TGF-β1+ si-SLC38A1+miR-150-5p inhibitor group or TGF-β1+ si-SLC38A1+ZFSA1 group) (Figure 6C, 6D). Moreover, western blot results showed that the protein levels of α-SMA, collagen I and FN1 were decreased in the TGF-β1+si-SLC38A1 group compared with the TGF-β1-treatment only group (P<0.01, Figure 6F, 6G), but the expression of E-cadherin was increased (P<0.01). Furthermore, immunofluorescence staining showed that silencing SLC38A1 restored the promoting effect of TGF-β1 treatment on the expression of α-SMA (Figure 6E), but upregulated the expression of E-cadherin (Figure 6H). However, the above effects were alleviated in the rescue group. Overall, knockdown of SLC38A1 significantly promoted TGF-β1-induced fibroblast activation.

**Figure 7:**
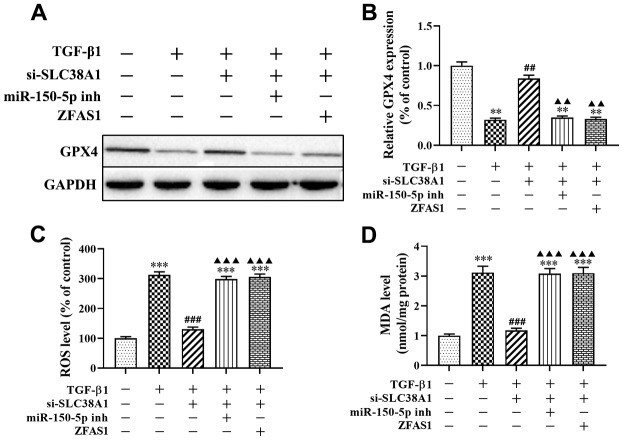
**lncRNA ZFAS1 affects TGF-β1-induced ferroptosis in HFL1 cells by regulating the miR-150-5p/SLC38A1 axis.** (**A**, **B**) Western blot was performed to detect the level of GPX4 protein; (**C**) the level of ROS was measured by ROS kit; (**D**) The MDA content was measured by lipid peroxidation (MDA) assay kit; ^**^P<0.01, ^***^P<0.001, compared with the control group; ^##^P<0.01, ^###^P<0.001, compared with the TGF-β1 group; ^▲▲^P<0.01, ^▲▲▲^P<0.001, compared with the TGF-β1+si-SLC38A1 group.

In addition, the effect of the loss-of function of si-SLC38A1 on ferroptosis in TGF-β1-treated HFL1 cells was investigated. Western blot analysis showed that SLC38A1 suppression significantly increased the protein level of GPX4 compared with that in the TGF-β1 group (P<0.01, Figure 7A, 7B). In addition, the ROS levels and MDA production were decreased when SLC38A1 knockdown in TGF-β1-treated HFL1 cells (all P<0.01, Figure 7C, 7D). However, there was no significant difference between the TGF-β1 treatment group and the rescue group (Figure 7). Taken together, the results showed that overexpression of lncRNA ZFAS1 increased the promoting effect of TGF-β1 on ROS levels and ferroptosis in HFL1 cells by downregulating the inhibitory effect of miR-150-5p on SLC38A1 expression.

### Knockdown of lncRNA ZFAS1 blocks BLM-induced PF via regulation of the miR-150-5p/SLC38A1 axis

**Figure 8:**
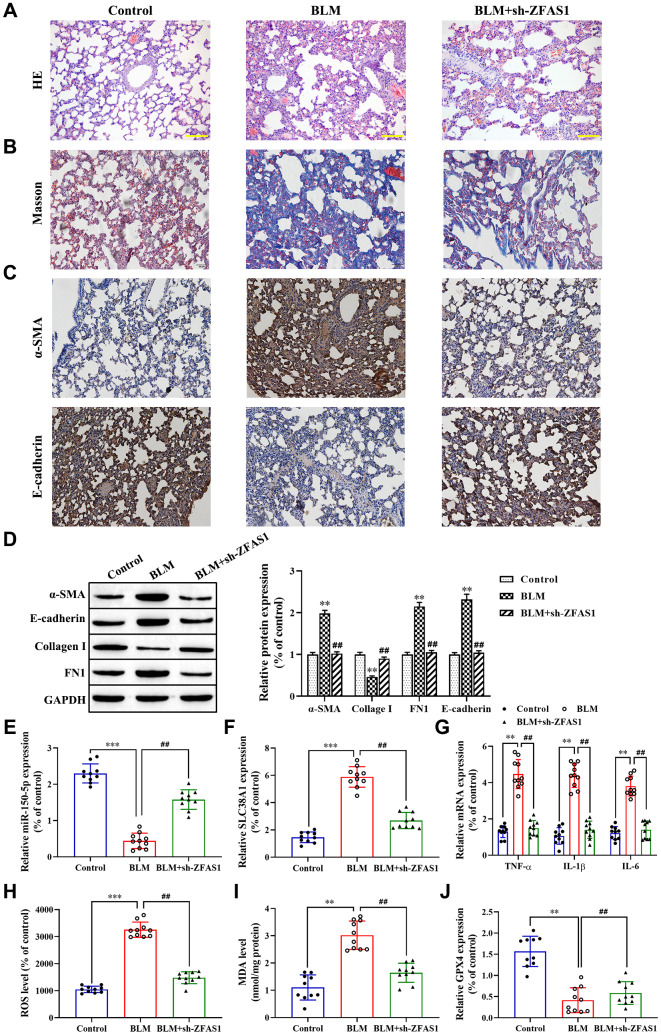
**Knockdown of lncRNA ZFAS1 blocks BLM-induced PF via regulation of the miR-150-5p/SLC38A1 axis.** (**A**, **B**) HE and Masson staining were used to observe the morphological changes of lung tissues; (**C**, **D**) Immunohistochemistry assay was performed to detect the expression of E-cadherin and α-SMA in lung tissues; (**D**) Western blot was performed to measure the expression of E-cadherin, collagen I, FN1 and α-SMA in lung tissues; (**E**–**G**) The expression of miR-150-5p, SLC38A1, inflammatory cytokines in lung tissues were determined by RT-qPCR; (**H**) the level of ROS was measured by ROS kit; (**I**) The MDA content was measured by lipid peroxidation (MDA) assay kit; (**J**) RT-qPCR was performed to detect the level of GPX4 mRNA. ^**^P<0.01, ^***^P<0.001, compared with the control group; ^##^P<0.01, compared with the BLM-treated group.

To further verify the role of the lncRNA ZFAS1/miR-150-5p/SLC38A1 axis in the progression of PF *in vivo*, H&E and Masson staining were performed. The results showed that BLM administration caused the thickening of pulmonary interalveolar septa, inflammatory cell infiltration, and increased collagen deposition compared with the control group (Figure 8A, 8B), but knockdown of lncRNA ZFAS1 attenuated this effect. Moreover, immunohistochemistry assay revealed that lncRNA ZFAS1-silenced decreased the expression of α-SMA in the lung tissues of BLM-induced PF rat model (Figure 8C), but enhanced E-cadherin (Figure 8C). Consistent with the immunohistochemistry assay results, the western blot results showed that lncRNA ZFAS1-silenced decreased the expression of α-SMA, collagen I and FN1 in the lung tissues of the BLM-induced rat model (all P<0.01, Figure 8D) and increased the expression of E-cadherin (P<0.01, Figure 8D). Besides, knockdown of lncRNA ZFAS1 significantly increased the expression of miR-150-5p in lung tissues of the BLM-induced PF rat model (P<0.01, Figure 8E), but decreased the mRNA expression of SLC38A1 (P<0.01, Figure 8F). Moreover, silencing of lncRNA ZFAS1 significantly decreased the expression of serum inflammatory cytokines in the BLM-induced rat model (all P<0.01, Figure 8G). Furthermore, the effect of lncRNA ZFAS1 on ROS levels and the expression of MDA and GPX4 in lung tissues were determined. The results showed that BLM administration significantly enhanced ROS levels and MDA generation compared with the control group (P<0.001, P<0.01, Figure 8H, 8I), but decreased GPX4 protein levels (P<0.01, Figure 8J). However, knockdown of lncRNA ZFAS1 reduced the inducing effect of BLM treatment on the lipid peroxidation and ferroptosis (all P<0.01, Figure 8H–8J). Overall, knockdown of lncRNA ZFAS1 significantly attenuated BLM-induced PF and ferroptosis by regulating the miR-150-5p/SLC38A1 axis.

## DISCUSSION

In this study, our results demonstrated that lncRNA ZFAS1 was highly expressed in lung tissues of BLM-induced PF rat model. Moreover, knockdown of lncRNA ZFAS1 significantly inhibited BLM-induced PF by suppressing the process of FMT and lipid peroxidation. Mechanistically, silencing of lncRNA ZFAS1 restricted BLM-induced PF and decreased migration and FMT of HFL1 cells treated with TGF-β1 by sponging miR-150-5p. Furthermore, knockdown of SLC38A1 significantly attenuated the TGF-β1-induced FMT process and lipid peroxidation. Of note, SLC38A1, a key positive regulator in lipid peroxidation, was found to be a target gene of miR-150-5p. Collectively, these results suggest that knockdown of lncRNA ZFAS1 ameliorated BLM-induced PF by lncRNA ZFAS1 acting as a ceRNA and sponging miR-150-5p to downregulate SLC38A1 expression, which provides a new therapeutic target for the treatment of PF.

Accumulating evidence has confirmed that lncRNAs are long RNA transcripts without protein-coding ability and are widely involved in various aspects of cellular processes [31, 32]. Previous studies suggest that the abnormal expression of lncRNA was closely related to the development and prognosis of many human diseases [33, 34]. Hence, dysregulated lncRNA may function as an important biological marker for various diseases including PF. lncRNAs exert their functions in different ways, such as modification of transcription, translation and post-translational levels. Recently, lncRNAs have attracted extensive attention as a ceRNA that binds to miRNA through a “sponge” adsorption mechanism [35]. Thus, in addition to miRNAs mediating mRNA degradation and transcriptional inhibition, lncRNAs could reversely regulate miRNAs by competing with mRNAs for binding to miRNAs. At present, the role of lncRNA as a ceRNA in the biological behaviors of human cancer cells has been reported broadly [36, 37]; however, only a few studies have reported a similar role of lncRNA in PF. For example, Yan et al. found that lncRNA MALAT1 promoted silica-induced PF by sponging miR-503 to upregulate the expression of PI3K [38]. Qian et al. confirmed that lncRNA ZEB1-AS1 promoted BLM-induced PF by ZEB1-mediated EMT via competitively binding miR-141-3p [39]. Li et al. found that lncRNA RFAL accelerated PF progression by CTGF through competitively binding miR-18a [40]. In the present study, we for the first time to explore the role of lncRNA ZFAS1 in the progression of PF. As expected, our data revelated that lncRNA ZFAS1 was overexpressed in lung tissues of BLM-induced PF, and overexpression of lncRNA ZFAS1 accelerated the progression of PF through sponging miR-150-5p to upregulate SLC38A1 expression. In addition, previous studies confirmed that lncRNA ZFAS1 acts as an oncogene gene to promote the growth and metastasis of multiple solid tumors by mediating the EMT and proliferation of cancer cells [30, 41].

In recent years, abnormal expression of ROS has been associated with several diseases, including PF [42], and ROS plays an important role in regulating cell apoptosis, differentiation and viability. For example, high levels of ROS could promote TGF-β1-induced fibrosis and FMT process [43]. Similarly, ROS-mediated oxidative damage through upregulation of inflammatory cytokine secretion in the progression of PF [44, 45]. Besides, the ROS level acts as an important marker of lipid peroxidation, and lipid peroxidation leads to ferroptosis by up-regulating ROS levels in PF [16]. Similar results have been reported in that the level of ROS was elevated in TGF-β1-induced HFL1 cells, but treatment with ferroptosis inhibitor abolished this effect [21]. In the present study, our results showed that treatment with TGF-β1 significantly increased the level of ROS and inflammatory cytokines, but Fer-1 administration or lncRNA ZFAS1 knockdown alleviated its expression.

Published studies have shown that miRNAs target downstream genes to alleviate the progression of PF by affecting ROS levels [46]. For example, Fierro-Fernández et al. found that miR-9-5p suppressed ROS levels, pro-fibrogenic transformation and fibrosis through targeting NOX4 and TGFBR2 [47]. In addition, previous studies revealed that miRNAs play an important role in regulating the FMT process, fibroblast apoptosis and inflammation to mediate the progression of PF [48–51]. In this study, our data showed that knockdown of miR-150-5p significantly promoted the expression of inflammatory cytokines, fibroblast activation and ROS levels by targeting SLC38A1 expression.

Ferroptosis is a lipid- and iron-dependent form of cell death that is different from cell apoptosis, pyroptosis and autophagy. Recently, studies found that ferroptosis biomarkers (ROS, MDA and GPX4) have been detected in the tissues of fibrosis-related diseases [52–54]. In the present study, we found that the expression of ROS and MDA were upregulated in HFL1 cells treated with TGF-β1, but GPX4 expression was decreased. However, treatment with Fer-1 alleviated the promotion effect of TGF-β1 on lipid peroxidation. Moreover, other studies confirmed that upregulation of GPX4 against the TGF-β1-induced FMT process and oxidative stress [16, 21]. Besides, it has been demonstrated that the regulation of the glutamate/cystine antiporter system X_c_^–^ key proteins (SLC38A1, SLC1A5, SLC3A2 and SLC7A11) could also block ferroptosis in cells, for instance, miR-137 targets SLC1A5 to decrease ferroptosis [55] and upregulation of SLC7A11 promoted ferroptosis [56]. In this study, we confirmed that SLC38A1 was upregulated in lung tissues of the BLM-induced PF rat model. Silencing of SLC38A1 inhibited the FMT process, lipid peroxidation and inflammatory cytokine secretion.

In summary, we found that overexpression of lncRNA ZFAS1 and SLC38A1 were positively correlated with PF progression. Further study revealed that knockdown of lncRNA ZFAS1 significantly decreased fibroblast activation, lipid peroxidation, inflammation, and PF as a ceRNA to downregulate SLC38A1 by sponging miR-150-5p. Therefore, our findings suggest that the lncRNA ZFAS1/miR-150-5p/SLC38A1 axis plays an important role in the development and progression of PF, and may provide a novel biomarker for the diagnosis and prognosis of PF.

## MATERIALS AND METHODS

### Animal model

Male Sprague-Dawley rats (200-220 g) were purchased from Weitonglihua Company (Beijing, China) and maintained in a pathogen-free facility. All animal experiments were reviewed and approved by the Animal Ethics Committee of Kunming Medical University. After one week of adaptive feeding, a total of 30 rats were randomly divided into 3 groups (n=10 rats/group): a control group; bleomycin (BLM) group; and BLM+sh-ZFAS1 group. Rats in the BLM group were administered 5 mg/kg BLM (Nippon Kayaku, Japan) dissolved in phosphate buffered saline (PBS) and administered to the rats intratracheally to establish the PF model. Rats in the control group were treated with 0.05 mL PBS. Rats in the BLM+sh-ZFAS1 group were injected intraperitoneally with 30 μL lncRNA ZFAS1 shRNA adeno-associated virus 5 (Vigene Biosciences, USA) for 3 weeks prior to an injection of 5 mg/kg BLM sulfate. After 4 weeks, all rats were sacrificed, and lung tissues were collected for further experiments. Immunohistochemical staining was performed to observe the histomorphology and examine the expression of E-cadherin (1:1000, ab1416, Abcam, UK) and α-SMA (1:2000, ab32575, Abcam, UK) according to the previous studies [39]. Hematoxylin-Eosin (H&E) staining and Masson staining were performed to observe the morphological changes of lung tissues according to our previous studies [57].

### Cell culture and transfection

Human fetal lung fibroblast 1 (HFL1) cells were purchased from the Shanghai Institutes for Biological Sciences of the Chinese Academy of Sciences and cultured according to the manufacturer’s instructions. Media were supplemented with 1% penicillin-streptomycin and 10% FBS (Gibco; Thermo Fisher Scientific, Inc.) at 37 °C and 5% CO_2_. Subsequently, the cultured cells were randomly divided into three groups after culturing for 12 h. The TGF-β1-treated group were added with TGF-β1 (6 ng/mL; Sigma Aldrich, USA). The control group was added with the same amount of solvent. The TGF-β1+Ferrostain-1 (Fer-1)-treated group were treated with Fer-1 (1 μM; Sigma Aldrich, USA) prior to TGF-β1 treatment.

A total of 24 h prior to transfection, HFL1 cells were seeded in 6-well plates at the optimum density and incubated overnight. Next, sh-lncRNA ZFAS1, sh-SLC38A1, miR-150-5p mimics/inhibitor, and control were transfected into HFL1 cells using Lipofectamine^®^ 3000 reagent and Opti-MEM medium (Invitrogen; Thermo Fisher Scientific, Inc.) according to the manufacturer’s protocols. The sh-lncRNA ZFAS1 (sh-ZFAS1), SLC38A1 siRNA (si-SLC38A1), miR-150-5p mimics/inhibitor and blank plasmids were purchased from GenePharma (Shanghai, China).

### RT-qPCR analysis

The nuclear and cytoplasmic fractions were isolated using NE-PER^TM^ Nuclear and Cytoplasmic Extraction Reagents (Thermo Fisher Scientific, USA) according to the manufacturer’s instructions. Total RNA was isolated from cultured tissues and cells using TRIzol reagent (Qiagen GmbH) and revere-transcribed into cDNA using PrimeScript^TM^ RT Reagent Kit with a gDNA Eraser (Takara Bio, Inc.). Subsequently, qPCR analysis was performed in an Applied Biosystems 7500 Real Time PCR system (Thermo Fisher Scientific, USA) using SYBR Green PCR Master Mix (Takara, Japan) using the following conditions: Initial activation step at 95 °C for 5s, 35 cycles of denaturation at 94 °C for 15 s, annealing at 55 °C for 25 s, and extension at 70 °C for 30 s. The sequences of the primers for qPCR are presented in Table 1. U6 and GAPDH were used as internal controls. The 2^-∆∆Ct^ method was used to calculate the relative expression of IL-6, IL-1β, TNF-α, GPX4, lncRNA ZFAS1, miR-150-5p and SLC38A1. qPCR was performed in triplicate

**Table 1:** Name and sequences of the primers.

**Name**	**Primer sequences (5’-3’)**
lncRNA ZFAS1	F: CCGGAGTGTGGTACTTCTCC
	R: CCAGAGGTCTCCAACGAAGA
SLC38A1	F: GCTTTGGTTAAAGAGCGGGC
	R: CTGAGGGTCACGAATCGGAG
GPX4	F: TAAGAACGGCTGCGTGGTGAAG
	R: AGAGATAGCACGGCAGGTCCTT
miR-150-5p	F: CATGGCCCTGTCTCCCAAC
	R: GGCCTGTACCAGGGTCTGA
TNF-α	F: AGCCCCCAGTCTGTATCCTT
	R: CTCCCTTTGCAGAACTCAGG
IL-6	F: GCCCAAACACCAAGTCAAGT
	R: TATAGGAAACAGCGGGTTGG
IL-1β	F: CAGAAGTACCTGAGCTCGCC
	R: AGATTCGTAGCTGGATGCCG
U6	F: GCTTCGGCAGCACATATACT
	R: GTGCAGGGTCCGAGGTATTC
GAPDH	F: AAGGTCGGTGTGAACGGATT
	R: TGAGTGGAGTCATACTGGAACAT

F: Forward primer; R: Reverse primer.

### Western blot analysis

The PVDF membranes were incubated overnight at 4 °C with primary antibodies SLC38A1 against (1:1000, ab134268, Abcam, UK), E-cadherin (1:1000, ab1416, Abcam, UK), α-SMA (1:2000, ab32575, Abcam, UK), Fibronectin-1 (FN1) (1:1500, ab45688, Abcam, UK), collagen I (1:1000, ab34710, Abcam, UK), and GPX4 (1:1000, ab125066, Abcam, UK). The horseradish peroxidase-coupled secondary antibody (1:2000, ab182018, Abcam, UK) was incubated with the membranes for 1 h at room temperature. The ECL western blot detection kit (Bio-Rad, USA) was employed to detect the optical density of the protein bands.

### BrdU staining

Cells were inoculated into 96-well plates with 1×10^4^ cells per well and cultured in a 37 °C incubator with 5% CO_2_ for 1 h to allow cells to adhere. Then, the cells were incubated with 5-bromodeoxyuridine (BrdU) for 1 h and stained with anti-BrdU (ab1893, Abcam, UK) following the manufacturer’s protocol. All stained images were observed and imaged with a scanning microscope (Olympus, Japan).

### Wound healing assay

HFL1 cells (1.5×10^6^ cells/well) were treated with different reagents, seeded in six-well plates and cultured until they reached confluence. Wounds were made in the cell monolayer by making a scratch with a 20 μL pipette tip. The plates were washed once with fresh medium after 24 h in culture to remove non-adherent cells. Following this wash, the cells were imaged. The migration distance of cells were measured according to the following formula: Migration rate (%)=(W_0h_-W_24h_)/W_0h_×100%.

### Immunofluorescence staining

Cells were seeded onto glass slides beforehand. When the confluence reached 70%, the cells were fixed with 4% paraformaldehyde (Sinopharm, China) for 15 min and permeabilized with 0.1% Triton X-100 (Amresco, USA) at room temperature for 30 min. After blocking with goat serum (Solarbio, China) for 15 min, the cells were incubated with rat anti-α-SMA (1:2000, ab32575, Abcam, UK) and rat anti-E-cadherin (1:1000, ab1416, Abcam, UK) at 4°C overnight. After rinsing with PBS, the cells were incubated with goat anti-rabbit IgG labeled with Cy3 (1:500, ab6939, Abcam, UK) at room temperature in the dark for 1 h. After being counterstained with 4’,6-diamidino-2-phenylindole (DAPI, Invitrogen, USA), cells were mounted in the presence of anti-fluorescence quenching agent (Abcam, UK), observed and imaged with a fluorescence microscope (Olympus, Japan).

### Fluorescence in suit hybridization (FISH)

Cells were incubated with 0.2 mol/L HCl for 0.5 h after fixation with 4% formaldehyde for 20 min, and then incubated with 5 μg/mL proteinase K for 15 min. Acetylated in a specific solution, and hybridized with FITC labeled lncRNA ZFAS1 probe (5 μg/mL) for 1 day. Subsequently, the cells were washed twice with 2× SSC detergent containing 0.01% Tween-20 at 55 °C. Afterwards, FITC-labeled probes were detected using standard immunofluorescence protocols.

### RNA immunoprecipitation (RIP)

The RIP assay was conducted using the Magna RIP RNA-binding protein immunoprecipitation kit (Millipore, USA) according to the manufacturer’s protocol. RIP was performed using HFL1 cell lysate and either anti-Ago2 (ab32381, Abcam, UK) or Rabbit IgG (ab172730, Abcam, UK) as the antibody. Subsequently, RT-qPCR was used to detect the expression of purified RNA.

### Determination of MDA generation

The tissues or cells were mixed with PBS at a 9-fold volume, dispersed into a single cell suspension, freeze-thawed for 3 times, and centrifuged in 12000 rpm for 10 min. The supernatant was collected as the protein sample, and the concentration was detected by BCA protein quantitative kit (Beyotime Biotechnology, China) according to the protocol. MDA content was detected by a Lipid Peroxidation (MDA) assay kit (ab118970, Abcam, UK) according to the manufacturer’s protocol.

### Determination of the ROS level

The OxiSelectIn Vitro ROS/RNS Assay Kit (Cell Biolabs, USA) was used to detect the level of ROS in the lung tissues and cell samples. The ROS level in each group was analyzed in triplicate using commercial kits according to the manufacturer’s instructions.

### Dual-luciferase reporter gene assay

The cells were seeded in 24-well plates at a density of 60%. According to the manufacturer’s instructions, the reporter construct containing the lncRNA ZFAS1 wild-type (WT) or mutant (MUT) 3’UTR was co-transfected into cells with miR-150-5p using Lipofectamine 3000 reagent. After 48 h, the cells were collected and tested for luciferase by a Dual-Luciferase Assay System (Promega, USA). The target verification methods for SLC38A1 and miR-150-5p were similar to those mentioned above.

### Statistical analysis

All data were collected and presented as the mean ± standard deviation. The data were then analyzed by using SPSS 22.0 software (IBM, USA). Spearman correlation analysis was performed to analyze the correlation among lncRNA ZFAS1, miR-150-5p and SLC38A1 in lung tissues of the PF model using Graphpad Prism (Version 8.0.2). P<0.05 was considered to indicate a statistically significant difference.
